# Corneal thickness evaluation in healthy eyes: Comparison between two different Scheimpflug devices

**DOI:** 10.1371/journal.pone.0243370

**Published:** 2020-12-03

**Authors:** Nicola Rosa, Maddalena De Bernardo, Angela Pepe, Livio Vitiello, Giuseppe Marotta, Roberto Imparato, Luigi Capasso

**Affiliations:** 1 Department of Medicine, Surgery and Dentistry, “Scuola Medica Salernitana” University of Salerno, Baronissi, Salerno, Italy; 2 Corneal Transplant Unit, ASL Napoli 1, Naples, Italy; Universidad de Monterrey Division de Ciencias de la Salud, MEXICO

## Abstract

**Purpose:**

To evaluate the correlation between corneal thickness (CT) measurements obtained with two Scheimpflug devices, Pentacam HR and Precisio, and to elaborate, if necessary, a regression formula which could make these results comparable.

**Design:**

Retrospective, Comparative, Observational study.

**Setting:**

Department of Medicine, Surgery and Dentistry, “Scuola Medica Salernitana” University of Salerno, Italy

**Methods:**

One hundred twenty four healthy eyes of 124 volunteers (65 males; range: 20–32 years; mean age of 24.8 ± 1.7) were included in this study. CT was measured using Pentacam HR and Precisio in three different points: the pupil center (PC), the corneal apex (CA) and the thinnest point (TP).

**Results:**

CT obtained with both devices at the PC, at the CA and at the TP showed a good correlation (r = 0.97, r = 0.97, r = 0.97, respectively), but Pentacam HR measurements were significantly thicker than those provided by Precisio (p < 0.01). The differences between Pentacam HR and Precisio were 21.9 ± 8.8 μm at the PC, 21.9 ± 8.9 μm at the CA, 19.1 ± 9.0 μm at the TP. The calculated regression formulas were: y = 0.9558x + 2.3196 for the PC, y = 0.9519x + 4.5626 for the CA, y = 0.9364x + 15.436 for the TP, where x is the CT measured with Pentacam HR and y is the Precisio measurement.

**Conclusions:**

The findings provided by this study highlight that Precisio measures thinner corneas compared to Pentacam HR. The identified regression formulas could be utilized to make interchangeable the results obtained with these two devices.

## Introduction

In recent years, an accurate measurement of corneal thickness (CT) or pachymetry has aroused an ever increasing interest, as a good indicator of the corneal health state [1]. Moreover, there are numerous fields in which clinical use of CT occurs, such as corneal refractive surgery [2–4], evaluation of intraocular pressure (IOP) [5, 6], endothelial cells function and health [7], and the discrimination of keratoconus from contact lens induced corneal thinning [8].

There are several types of pachymetry based on different methods of CT measurements, such as dual beam partial coherence interferometry (PCI), optical pachymetry with rotating Scheimpflug camera (Pentacam), optical coherence tomography (OCT), contact and non-contact specular microscopy (SM), ultrasound pachymetry (UP) and ultrasound biomicroscopy (UBM), slit scanning corneal tomography (Orbscan), confocal microscopy.

Nowadays, some people consider UP the gold standard for the CT assessment; this technique has the advantage of using a portable instrument, but the limitations are represented by the need for topical anesthesia [9], risk of infection or contact corneal trauma, considerable operator skill and often unrepeatable probe placement [10, 11]. Inversely, optical pachymetry with rotating Scheimpflug camera is a non-contact method which does not need anesthesia, it has no risk of corneal infections, it is very comfortable for the patient and it is less operator dependent [12, 13].

The aim of this study was to evaluate the correlation between CT measurements provided by two different Scheimpflug devices, Pentacam HR (Oculus, Wetzlar, Germany) and Precisio (iVis Technologies, Taranto, Italy), and to elaborate, if necessary, a regression formula which could make these results comparable.

## Materials and methods

### Patients selection

One hundred twenty four eyes of 124 volunteers (65 males) with a mean age of 24.8 ± 1.7 (range: 20–32 years) were included in this retrospective, observational study. Subjects with systemic and ocular diseases (such as keratoconus, corneal ectasias and opacities) and patients with a history of previous refractive surgery were excluded. Each subject was also asked to stop wearing contact lenses at least three days before the exams.

The volunteers were informed about the purpose of the study and a written informed consent, according to the ethical principles of the Declaration of Helsinki, was acquired. Institutional Review Board approval was also obtained (CECS, Cometico Campania Sud).

### Devices characteristics and measurements acquisition

CT measurements were randomly performed on the same day by different operators using two tomographs, Pentacam HR and Precisio, both based on Scheimpflug’s principle. For each volunteer, only the right eye was evaluated.

Pentacam HR is a combined device consisting of a slit illumination system (blue led at 475 nm) and a Scheimpflug camera, which rotate together around the optical axes of the eye. Within 2 seconds, the system generates 50 sectional images of corneal surface analyzing 500 measurement points for each single image (50 x 500 = 25000 points).

Precisio is a new generation corneal tomograph. Its image acquisition system is based on 2 gigabit complementary metal-oxide semiconductor digital cameras. It has a blue scanning laser micro-slit which allows to register and validate epithelial maps up to 10.0 mm diameter, acquiring over 120000 points for each analyzed surface. Moreover, Precisio provides 60 high definition cross-section images in less than 1 second using a 3D tracking, offering morphological and refractive data of each corneal sublayer.

During the execution of both the exams, the volunteers were asked to seat in front of the device, with chin and forehead resting on the appropriate supports, to keep both eyes open and to fixate on a blinking fixation target in the camera’s center. The operator visualized the image of the patient's eye on a computer screen and focused it by moving the joystick of the instrument. As soon as the image was perfectly aligned, the patient was asked not to move and to keep eyes open, so the scan was started.

The authors compared the CT values obtained with Pentacam HR and Precisio in three different points: the pupil center (PC), the corneal apex (CA) and the thinnest point (TP).

### Statistical analysis

All data were entered into a Microsoft Excel spreadsheet; mean, standard deviation, maximum and minimum values for each parameter set were calculated. The data were analyzed using three scatter plots (one for each point where the CT was measured), reporting CT values measured with Pentacam HR on the x axis and those ones measured by Precisio on the y axis.

The Student paired T test and Pearson correlation coefficient (r) were used to calculate the level of statistical significance and the correlation between the two methods [14, 15]. Reliability and agreement between Pentacam HR and Precisio were defined with MedCalc 19.1 (Mariakerke, Belgium), using intraclass correlation coefficient (ICC), Bland-Altman plots and 95% confidence interval of limits of agreement [16, 17]. P values less than 0.01 were considered statistically significant.

The sample size was determined by maximizing the statistical power. The analysis was performed by using G*Power 3.1 software [18]. A difference between two dependent means (matched pairs) was computed. Input data were the following: α was set at 0.01; 1-β was set at 0.95; effect size was set as medium at around 0.385. Results were the following: non-centrality parameter δ = 4.287; critical t = 2.616; Df = 123; actual power = 0.951; total sample size = 124.

## Results

CT values obtained with both Pentacam HR and Precisio are summarized in Table 1, while ICC and limits of agreement between the two devices are shown in Table 2.

**Table 1:** Corneal thickness measurements (in microns) obtained with Pentacam HR and Precisio at the pupil center, at the corneal apex and at the thinnest point expressed as mean, standard deviation, minimum and maximum values.

	PENTACAM HR			PRECISIO		
	PC (μm)	CA (μm)	TP (μm)	PC (μm)	CA (μm)	TP (μm)
**MEAN**	549.5	550.3	545.3	527.6	528.4	526.2
**SD**	35.5	35.7	35.5	35.0	35.1	34.7
**MIN**	456.0	455.0	453.0	432.0	432.0	431.0
**MAX**	657.0	663.0	655.0	644.0	643.0	643.0

PC: Pupil Center; CA: Corneal Apex; TP: Thinnest Point; SD: Standard Deviation; MIN: Minimum value; MAX: Maximum value.

**Table 2:** Intraclass correlation coefficient and limits of agreement (with their respective 95% confidence interval) for Pentacam HR and Precisio at the pupil center, at the corneal apex and at the thinnest point.

	ICC	95% CI	Upper limit	95% CI	Lower limit	95% CI
**PC**	0.97	0.96 to 0.98	+39.3	+36.6 to +42.0	+4.6	+1.9 to +7.3
**CA**	0.97	0.96 to 0.98	+39.3	+36.6 to +42.0	+4.5	+1.8 to +7.2
**TP**	0.97	0.95 to 0.98	+36.8	+34.1 to +39.6	+1.4	-1.4 to +4.1

ICC: Intraclass Correlation Coefficient; 95% CI: 95% Confidence Interval; PC: Pupil Center; CA: Corneal Apex; TP: Thinnest Point.

The Pentacam HR measurements were significantly thicker than those provided by Precisio (p < 0.01). The differences between Pentacam HR and Precisio were 21.9 ± 8.8 μm at the PC, 21.9 ± 8.9 μm at the CA, 19.1 ± 9.0 μm at the TP.

**Fig 1:**
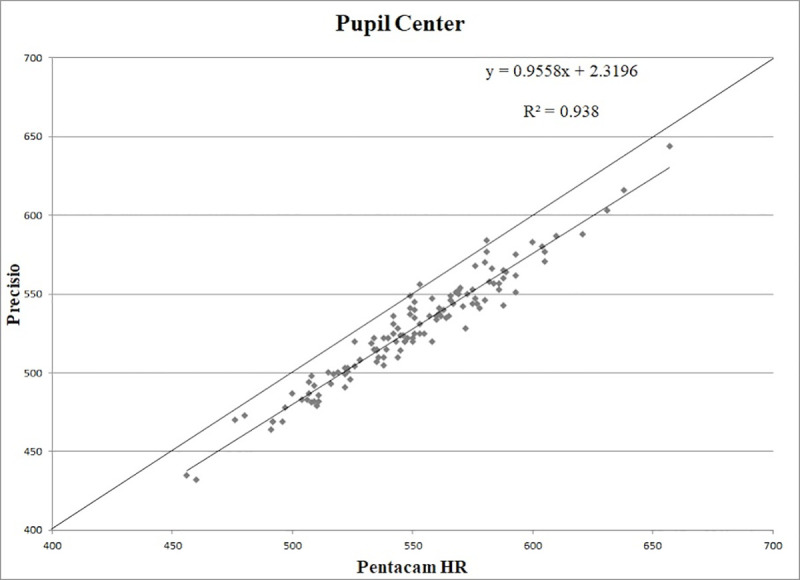
Scatter plot of correlation between corneal thickness measurements (in microns) obtained with Pentacam HR and Precisio at the pupil center. R^2^: determination coefficient.

**Fig 2:**
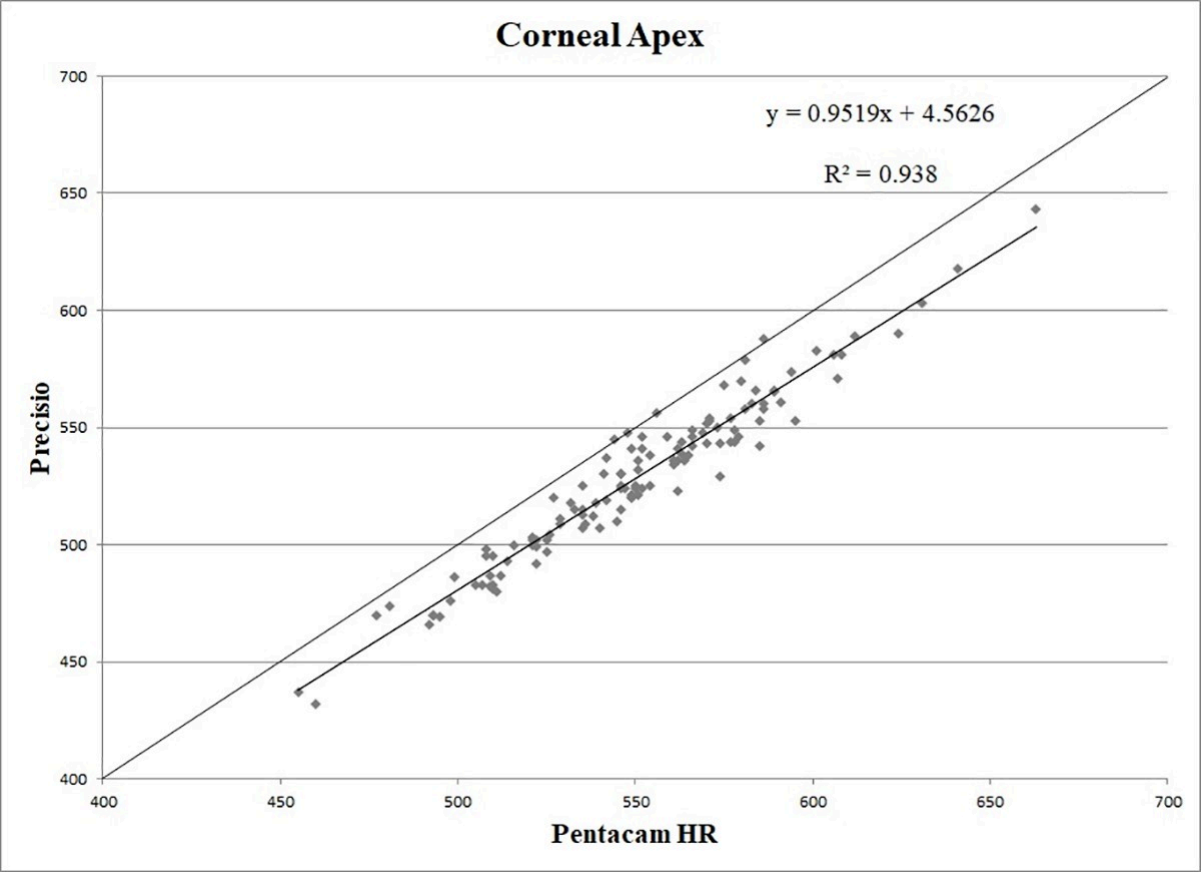
Scatter plot of correlation between corneal thickness measurements (in microns) obtained with Pentacam HR and Precisio at the corneal apex. R^2^: determination coefficient.

**Fig 3:**
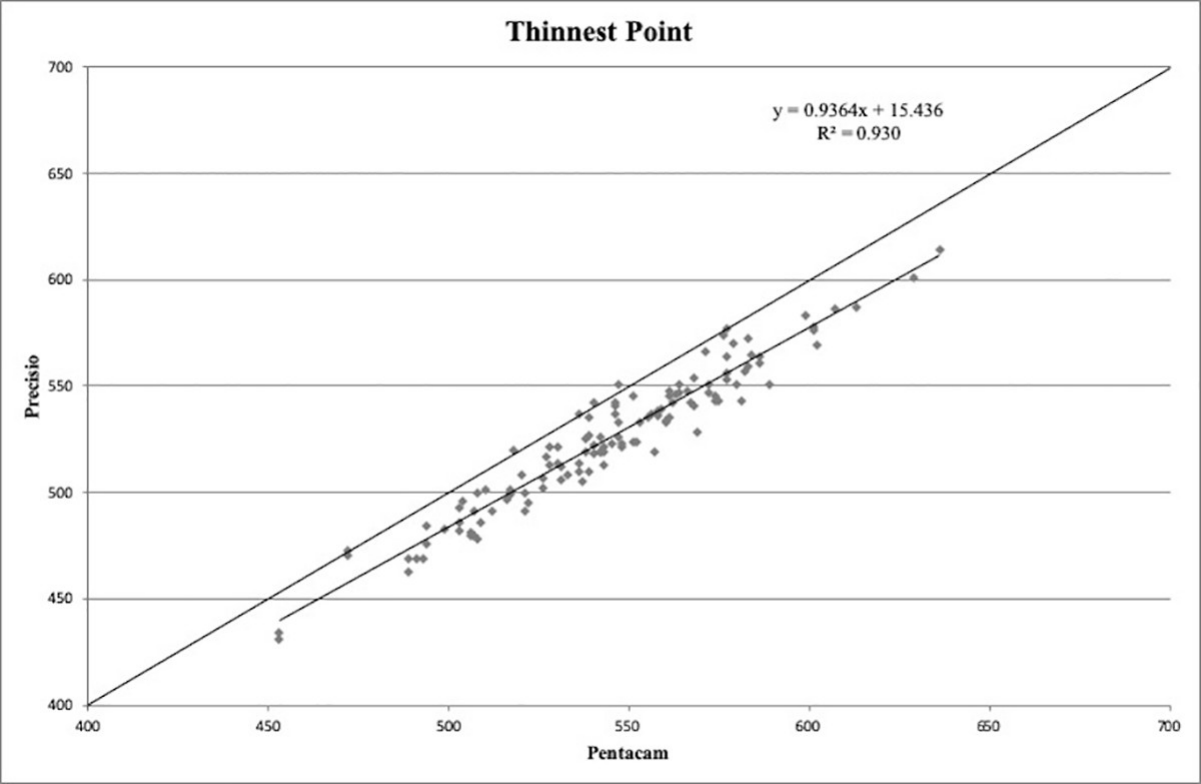
Scatter plot of correlation between corneal thickness measurements (in microns) obtained with Pentacam HR and Precisio at the thinnest point. R^2^: determination coefficient.

CT values obtained at the PC, CA and TP showed a statistically significant correlation (p < 0.01), with the following correlation coefficients: r = 0.97, r = 0.97, r = 0.97, respectively (Figs 1–3).

The good correlation between the measurements provided by the two Scheimpflug devices suggested to look for regression formulas that could make the results obtained with these two different tomographs comparable.

The calculated regression formulas were: y = 0.9558x + 2.3196 for the PC, y = 0.9519x + 4.5626 for the CA, y = 0.9364x + 15.436 for the TP, where *x* is the CT measured with Pentacam HR and y is the measurement obtained with Precisio.

**Fig 4:**
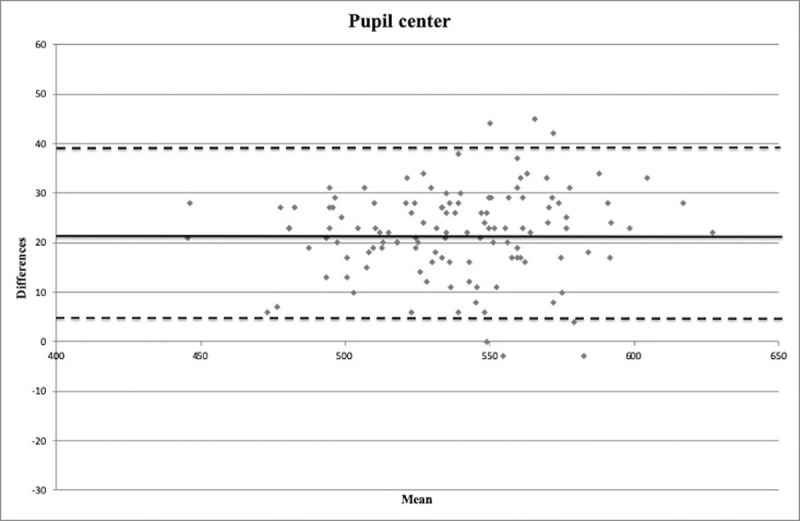
Bland-Altman plot between corneal thickness measurements (in microns) obtained with Pentacam HR and Precisio at the pupil center. Continuous line: mean difference (21.9 microns). Dashed lines: mean ± 1.96 standard deviation of the differences (4.6–39.3 microns).

**Fig 5:**
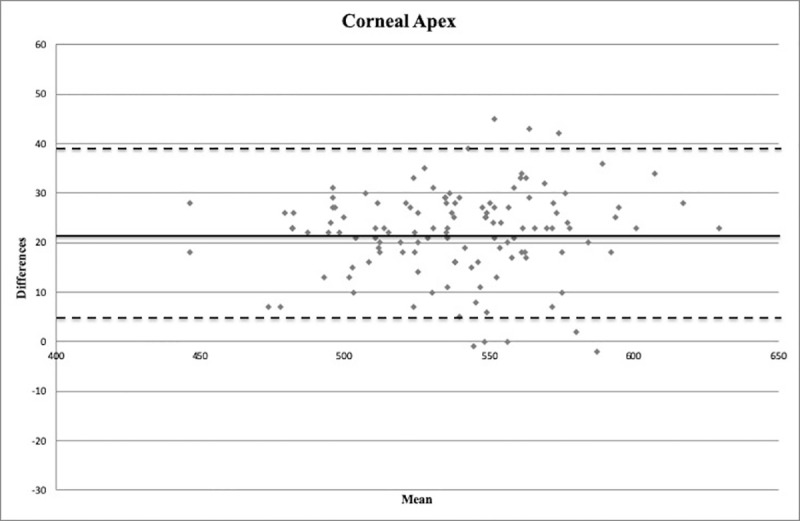
Bland-Altman plot between corneal thickness measurements (in microns) obtained with Pentacam HR and Precisio at the corneal apex. Continuous line: mean difference (21.9 microns). Dashed lines: mean ± 1.96 standard deviation of the differences (4.5–39.3 microns).

**Fig 6:**
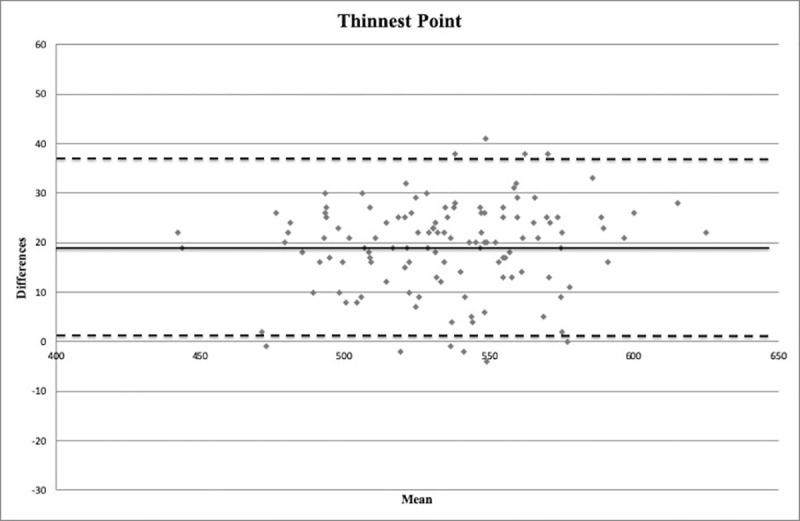
Bland-Altman plot between corneal thickness measurements (in microns) obtained with Pentacam HR and Precisio at the thinnest point. Continuous line: mean difference (19.1 microns). Dashed lines: mean ± 1.96 standard deviation of the differences (1.4–36.8 microns).

Bland-Altman plots are shown in Figs 4–6. An important information about the interchangeability between the two measurement methods is provided by the distribution of points around the mean value of differences and within the limits of the confidence interval.

## Discussion

CT measurement can be influenced by many different factors: age [19], familiarity and genetics [20, 21], circadian rhythms [22], contact lens wear [23], anthropometric characteristics [24, 25], antiglaucoma therapy [26, 27].

The importance of precise CT measurements to plan keratorefractive surgery and to diagnose and to follow-up glaucomatous patients, made the assessment of this parameter increasingly important in ophthalmological clinical practice [28–31].

The presence of a wide range of instruments able to measure CT makes the comparison of different devices essential. As it has been previously pointed out, ultrasound pachymetry is the most commonly used method and for a long time it has been considered the gold standard for measuring CT. However, this technique requires use of topical anesthetic drops, it is extremely user dependent and carries disease transmission risk. Due to these drawbacks, there is an increased market of "non-contact" CT measurement methods, including those based on Scheimpflug technology, which undoubtedly represent an important innovation in the assessment of the anterior segment.

In the present study, the authors compared CT measurements provided by Pentacam HR and Precisio to evaluate the agreement and the interchangeability between the two devices in the routine clinical practice.

To best of our knowledge, even if numerous studies have been carried out to compare different pachymetric methods, this is the first study evaluating the CT measurements provided by Precisio and another Scheimpflug device.

Baradan-Rafii et al [32] compared the anterior segment indices, including CT, measured by two Scheimpflug devices, Pentacam and Galilei in 176 eyes of 88 participants: mean central corneal thickness obtained with Galilei was significantly thicker than that one measured by Pentacam, with a range of 95% of limit of agreement (-62 to -31 μm) too wide. For this reason, the authors did not suggest to use the two systems interchangeably.

Anayol et al [33] compared the central corneal thickness and the thinnest corneal measurement among Galilei, Pentacam and Sirius in the right eye of 32 healthy subjects. They found that CT values obtained with Galilei were significantly higher than those ones obtained with either Sirius and Pentacam, and showed that Pentacam and Sirius had better agreement with each other than with Galilei. The authors concluded that these different devices cannot be used interchangeably.

Jahadi Hosseini et al [34] analyzed 47 eyes of 47 healthy subjects and found a good correlation and agreement among Galilei, Pentacam HR and Ultrasound Pachymetry in CT measurement. However, CT values obtained with Pentacam HR were lower than those obtained with Galilei.

Savini et al [35] compared central corneal thickness obtained with three Scheimpflug cameras (Pentacam, Sirius and TMS-5) in 25 eyes of 25 patients. Sirius and TMS-5 showed the worst agreement and the largest difference, with mean Sirius measurements 24 μm thicker than TMS-5. Sirius and Pentacam showed a poor agreement too, even if the differences were not statistically significant. TMS-5 and Pentacam showed a slightly better agreement. Thus, the authors suggested to be careful utilizing these devices interchangeably.

Lanza et al [36] compared corneal pachymetry values measured by three different devices, Orbscan II, Pentacam HR and Sirius in 102 healthy eyes. The difference found between Sirius and Pentacam in CT measurement did not appear to be statistically significant, in agreement with Huang et al [37], Anayol et al [33], and Savini et al [35]. The CT values provided by Orbscan II were lower than those provided by Pentacam HR and Sirius, in agreement with previous study by Rosa et al [38].

Huang et al [37] compared CT measurements using Pentacam, Sirius, Galilei and RTVue-100 OCT (Optovue Inc., USA) in 66 eyes of 66 healthy subjects revealing statistically significant differences among the four devices in CT measurements. Galilei showed the thickest CT values, RTVue OCT the thinnest ones, whereas Sirius and Pentacam were in between, with Sirius CT values thicker than Pentacam. The agreement between CT values provided by Sirius and Pentacam was good, whereas Galilei overestimated and RTVue underestimated CT compared to Sirius and Pentacam.

In the present study, the CT was found to be thicker with Pentacam HR than with Precisio, differently from the aforesaid studies, in which Pentacam provided thinner CT values than other Scheimpflug cameras. These results could be due to the different acquisition system operated by the two devices, as Pentacam HR analyzes 25000 points for each single acquisition, whereas Precisio is able to acquire over 120000 points for each analyzed surface. Furthermore, although a good correlation is provided by Pearson coefficient and ICC (Table 2), the Bland-Altmann plots show that there are wide limits of agreement (Figs 4–6).

The 95% limits of agreement provide an interval within which 95% of differences between measurements by the two methods are expected to lie. This method could determine if a new device could replace an old one, or if they could be interchangeable [14, 15]. However, although Bland-Altman plots are good and reliable statistical tools in determining the level of agreement between two different measurement methods, the effective and acceptable agreement will only derive from the clinical realm [14, 15].

In the present study, a mean difference of about 20 microns between the two devices at the three different measurement points, with the limits of agreement in a range of about 35 microns, was found. Such differences in CT evaluation could be particularly significant in some clinical areas of ophthalmology, such as corneal refractive surgery [39], IOP measurement, and diagnosis and management of corneal disease [40], where a few microns could make a difference. For this reason, the two evaluated devices cannot be used interchangeably, unless the detected regression formulas are used, making them comparable.

This study has some limitations. First, the authors studied only the CT measurements provided by these two instruments, for this reason further studies should be suggested for a full comparison of both the devices. Second, the authors analyzed only healthy eyes in this study, and further studies should be required to compare also patients with corneal diseases, such as keratoconus.

## Conclusions

In conclusion, this is the first paper to compare Precisio and Pentacam HR CT measurements, highlighting that Precisio measures thinner corneas compared to Pentacam HR.

It is not possible to state which device is more precise but, according to the studies that show thinner values provided by Pentacam HR than other Scheimpflug devices, Precisio seems to give thinner values than Pentacam HR.

The devices interchangeability could be very useful for planning corneal refractive surgery or during the follow-up of patients with keratoconus, that sometimes could be examined in different offices with different devices.

Finally, although the two devices provide different results, suggesting that in clinical practice they should not be used interchangeably, the regression formulas described in this study could support physicians to easily convert one measurement into another and to make them comparable.

## Supporting information

S1 Data (ZIP)
